# Anatomic single-bundle anterior cruciate ligament reconstruction using a calcium phosphate-hybridized tendon graft: a randomized controlled trial with 2 years of follow-up

**DOI:** 10.1186/s13018-018-1045-1

**Published:** 2018-12-29

**Authors:** Hirotaka Mutsuzaki, Tomonori Kinugasa, Kotaro Ikeda, Masataka Sakane

**Affiliations:** 1 Department of Orthopaedic Surgery, Ibaraki Prefectural University of Health Sciences, 4669-2 Ami Ami-machi, Inashiki-gun, Ibaraki 300-0394 Japan; 2 Department of Orthopaedic Surgery, Ichihara Hospital, 3681 Oozone, Tsukuba, Ibaraki 300-3295 Japan; 3 Department of Orthopaedic Surgery, Tsukuba Gakuen Hospital, 2573-1 Kamiyokoba, Tsukuba, Ibaraki 305-0854 Japan

**Keywords:** Anatomic single-bundle anterior cruciate ligament reconstruction, Calcium phosphate hybridization, Clinical result, Bone tunnel enlargement, A randomized controlled trial

## Abstract

**Background:**

To improve tendon-to-bone healing in anterior cruciate ligament (ACL) reconstruction, a novel technique via calcium phosphate (CaP)-hybridized tendon graft using an alternate soaking process was developed. The purpose of this study was to evaluate the clinical results of anatomic single-bundle ACL reconstruction using the CaP-hybridized tendon graft with up to 2 years follow-up, and compare the outcome with conventional ACL reconstruction and preoperative data.

**Methods:**

Ninety patients who required anatomic single-bundle ACL reconstruction were randomized to undergo either the CaP-hybridized tendon graft method (CaP group, *n* = 45) or the conventional method (conventional group, *n* = 45). At 1 and 2 years postoperatively, all patients were evaluated using KT-1000 arthrometry, pivot-shift test, International Knee Documentation Committee (IKDC) grade, Lysholm scale, and Tegner scale; at the same timepoints, bone tunnel enlargement was evaluated using computed tomography, and the tendon graft intensity was evaluated on magnetic resonance imaging. Tendon graft appearance was evaluated arthroscopically once after a period of up to 2 years postoperatively. Cases of re-rupture and adverse events were recorded in both groups.

**Results:**

In both groups, the KT-1000 arthrometry, pivot-shift test, IKDC grade, and Lysholm scale results at 1 and 2 years postoperatively were superior to preoperative data; these results did not significantly differ between groups at either timepoint. The rate of increase of the cross-sectional area of the femoral bone tunnel in the CaP group was smaller than that in the conventional group at 1 year postoperatively. The other results did not significantly differ between the two groups at any timepoint. There were two cases of re-rupture in the CaP group, and six cases of re-rupture in the conventional group. There were no adverse events during follow-up in either group.

**Conclusions:**

Anatomic single-bundle ACL reconstruction using a CaP-hybridized tendon graft was safe, and resulted in improved clinical outcomes at 2 years postoperatively compared with preoperative data; the outcomes were comparable with the conventional method. A longer follow-up is needed to clarify the clinical effects of the CaP-hybridized tendon graft in anatomic single-bundle ACL reconstruction.

**Trial registration:**

UMIN, UMIN000019788 Registered 14 November 2015—Retrospectively registered.

## Background

After anterior cruciate ligament (ACL) reconstruction using a soft tissue graft, only fibrous tissue that was mechanically inferior was noted between the grafted tendon–bone interface [1–3]. To improve tendon-to-bone healing, a novel technique via calcium phosphate (CaP)-hybridized tendon graft using an alternate soaking process was developed [4]. We consider that firm tendon-to-bone healing in ACL reconstruction can improve clinical results and prevent bone tunnel enlargement. Using the CaP-hybridized tendon graft, direct bonding between the grafted tendon and the newly formed bone without scar tissue formation at 2 to 3 weeks after ACL reconstruction in rabbits was observed [5, 6]. In the CaP group, better anterior knee stability and greater in situ forces in the graft under applied anterior tibial loads were found at 1 year after non-anatomic ACL reconstruction in goats compared with an untreated tendon graft [3]. In a clinical trial, the CaP-hybridized tendon graft in non-anatomic single-bundle ACL reconstruction improved anterior knee stability at 1 and 2 years postoperatively, and reduced the bone tunnel enlargement in both tunnels at 1 year postoperatively compared with the conventional method [7].

In anatomic single-bundle ACL reconstruction using the CaP-hybridized tendon graft, greater in-situ forces in the graft under applied anterior tibial loads were found at 6 months postoperatively in goats compared with an untreated tendon graft [8]. A clinical trial showed that using a CaP-hybridized tendon graft reduced bone tunnel enlargement on the femoral side at 1 year after anatomic single-bundle ACL reconstruction compared with an untreated tendon graft, and the clinical data were equal in both groups [9]. However, the clinical trial included bilateral ACL reconstruction cases, and the follow-up period was only 1 year. Therefore, it was not possible to accurately compare the clinical results.

Therefore, the purpose of the present study was to prospectively evaluate the clinical results of unilateral anatomic single-bundle ACL reconstruction using a CaP-hybridized tendon graft during 2 years of follow-up in comparison with the conventional method and with preoperative data. We hypothesized that anatomic single-bundle ACL reconstruction using the CaP-hybridized tendon graft would result in equivalent postoperative clinical results to the conventional method, and would reduce femoral bone tunnel enlargement compared with the conventional method.

## Methods

Between July 2011 and December 2015, a total of 90 patients who were scheduled to undergo arthroscopically assisted unilateral anatomic single-bundle ACL reconstruction using a hamstring tendon graft were enrolled in the present study. Patients were randomized to undergo either the CaP-hybridized tendon graft method (CaP group, *n* = 45) or the conventional method (conventional group, *n* = 45). The randomization was done according to the days of the week when the patients first visited the outpatient. All patients were followed up for a minimum of 2 years. The ethics committee of Ichihara Hospital reviewed and approved the study (approval number: 1101). Informed consent was obtained from the enrolled patients. The study protocol was registered with the University hospital Medical Information Network Clinical Trials Registry (UMIN000019788). We excluded revision cases, multi-ligamentous surgery cases, and bilateral ACL reconstruction cases. All ACL reconstructions were performed by two experienced surgeons (T.K. and H.M.). The patient characteristics did not differ between the two groups regarding age, sex, height, weight, operative findings of meniscal injury, and duration from injury to operation (Table 1). Meniscal pathology was confirmed by arthroscopic examination. In the CaP group, 4 medial and 6 lateral menisci were repaired, and 11 medial and 13 lateral menisci underwent partial resection; in the conventional group, 6 medial and 6 lateral menisci were repaired, and 1 medial and 12 lateral menisci underwent partial resection.

**Table 1:** Patient characteristics

	CaP group (*n* = 45)	Conventional group (*n* = 45)	*P* value
Age (years)	27.1 ± 11.1	22.9 ± 10.6	0.072
Sex (males/females)	21/24	26/19	0.294
Height (cm)	166.2 ± 8.3	167.3 ± 7.9	0.517
Weight (kg)	63.6 ± 11.2	66.3 ± 11.4	0.261
Operative findings of meniscal injury (MM/LM)	15/19	7/18	0.062
Duration from injury to operation (months)	7.9 ± 20.0	2.1 ± 2.2	0.057

The CaP group underwent single-bundle anterior cruciate ligament reconstruction with a calcium phosphate-hybridized graft, while the conventional group underwent reconstruction with a conventional tendon graft

Results are given as the mean ± SD

*MM* medial meniscus, *LM* lateral meniscus

### Surgical procedure

The surgical procedure was similar to that used in our previous study [9]. Briefly, after arthroscopic evaluation and treatment of associated lesions, the semitendinous tendon alone, or both the semitendinous and gracilis tendons, were harvested and used as multi-stranded grafts. The tendon graft was hooked to the TightRope RT® (Arthrex, Naples, FL, USA) on the femoral side. The free ends were whipstitched with FiberWire® #2 (Arthrex) on the tibial side. The length and diameter of the tendon grafts were 50–70 mm and 6.0–10.0 mm, respectively. Both the femoral and tibial bone tunnels were anatomically created at the tibial and femoral insertions of the ACL using the outside-in tunnel technique. A 5- to 15-mm-long femoral socket was created according to the length of the grafted tendon. The graft was fixed on the lateral femoral cortex, and then fixed to a screw and washer on the tibial side with an initial tension of 10 N using a tension meter at 20° of knee flexion.

### Intraoperative calcium phosphate hybridization method

The intraoperative CaP hybridization method was similar to that used in our previous study [7, 9]. After graft preparation, the intraarticular portion of the tendon graft was covered with the sleeve of a rubber glove tied on each side with nonabsorbable sutures to prevent CaP hybridization of the intraarticular portion [7, 9]. Then, the tendon graft was soaked in a calcium solution (100 mM CaCl_2_ + 30 mM l-histidine, pH 7.4, 280 mOsm/l, 20 °C) for 30 s. The grafts were subsequently soaked in a NaHPO_4_ solution (116.4 mM NaH_2_PO_4_:128.7 mM Na_2_HPO_4_·12H_2_O = 15%:85%, pH 7.4, 280 mOsm/l, 20 °C) for 30 s. Before each soaking, the grafts were washed in a saline solution. This cycle was repeated ten times [7, 9].

### Postoperative rehabilitation

Postoperative rehabilitation was similar to that performed in our previous study [9]. After surgery, the knee was immobilized at 20° flexion with a removable postoperative brace for 1 week. Range of motion exercise and partial weightbearing was started at 1 week postoperatively, and full weightbearing walking was allowed at 3 weeks postoperatively. Running was allowed at 3 months postoperatively, and return to sports was allowed after 6–12 months. The postoperative rehabilitation was the same for all patients, even those who received partial meniscectomy or meniscal repair.

### Clinical evaluations

The patients were assessed preoperatively, and at 1 and 2 years postoperatively. Anterior knee laxity was assessed using the KT-1000 arthrometer (MEDmetric, San Diego, CA, USA) at manual maximum anterior tibial load. The pivot-shift test, the Tegner scale [10], the International Knee Documentation Committee (IKDC) grade [11], and the Lysholm scale [10] were also assessed. Preoperative Tegner scale data were assessed using data obtained before ACL injury. Cases of re-rupture and adverse events (including infection, fracture, cancer, severe pain, contracture, etc.) were recorded in both groups.

### Computed tomography analysis

All patients underwent computed tomography (CT) evaluation at postoperative 1 week, 1 year, and 2 years. The full evaluation was performed independently by a radiologist who was blinded to the study grouping. CT scans (voltage 80 kV; Activion 16; Toshiba Medical Systems, Otawara, Japan) were performed with the knee in full extension to assess the femoral and tibial bone tunnels. Initial volume acquisition was made with 2-mm cuts from 10 cm above the femoral tunnel to 10 cm below the tibial tunnel. Three-dimensional images were reconstructed using a Virtual Place Lexus workstation (AZE, Tokyo, Japan) [7, 12, 13]. As the joint aperture site of the bone tunnels is reportedly enlarged after ACL reconstruction [12, 13], we evaluated both the femoral and the tibial tunnels at the aperture. The two groups were compared regarding the tunnel enlargement rates of the cross-sectional areas (CSAs) at the apertures of both the femoral and tibial tunnels from 1 week to 1 year postoperatively, and from 1 week to 2 years postoperatively.

The bone tunnel enlargement evaluation was performed using a similar method to that used in our previous study [9]. The tunnels were cut along planes perpendicular to the long axes. We measured the tunnel CSAs of the femur and tibia at the sites closest to the joint aperture. The increase in tunnel CSA was calculated as follows: CSA increase rate (%) = (CSA at 1 year or 2 year − CSA at 1 week) × 100 / CSA at 1 week.

### Magnetic resonance imaging evaluation

The magnetic resonance imaging (MRI) evaluation was similar to that described previously [7]. MRI evaluation was performed to analyze the intensity of the tendon graft, which is a measure of graft remodeling, at 1 and 2 years postoperatively. The tendon grafts of the mid-substance area were characterized as low intensity, isointensity, or high intensity by proton density imaging in the sagittal, coronal, and axial planes with 3.0-Tesla MRI (MAGNETOM Verio Dot; Siemens Healthineers, Earlangen, Germany) [7, 14]. The numbers belonging to each characterization were investigated and compared between both groups.

### Arthroscopic evaluation

The arthroscopic evaluation method was similar to that used in our previous report [7]. Second-look arthroscopic examination was performed once at 1 to 2 years postoperatively to analyze the synovium coverage of the tendon graft, which provides an indication of revascularization. The synovium coverage of each reconstructed graft was graded as A (completely covered), B (partially covered), or C (almost uncovered) [7, 15]. The numbers belonging to each grade were investigated and compared between both groups.

### Statistical analyses

The Student’s *t* test was used to analyze the results of KT-1000 arthrometry, Lysholm scores, and CT analyses between each group at the same timepoint. The chi-square for independence test was used to analyze the results of the pivot-shift test, Tegner scores, IKDC results, MRI evaluation, and arthroscopic appearance between groups at the same timepoint. The paired *t* test was used to analyze the KT-1000 arthrometry and Lysholm scores between preoperatively and 1 year postoperatively, and between preoperatively and 2 years postoperatively within the same group. The Wilcoxon signed-ranks test was used to analyze the pivot-shift test results, Tegner scores, and IKDC results between preoperatively and 1 year postoperatively, and between preoperatively and 2 years postoperatively within the same group. Differences were considered significant at *P* < 0.05.

## Results

**Fig. 1:**
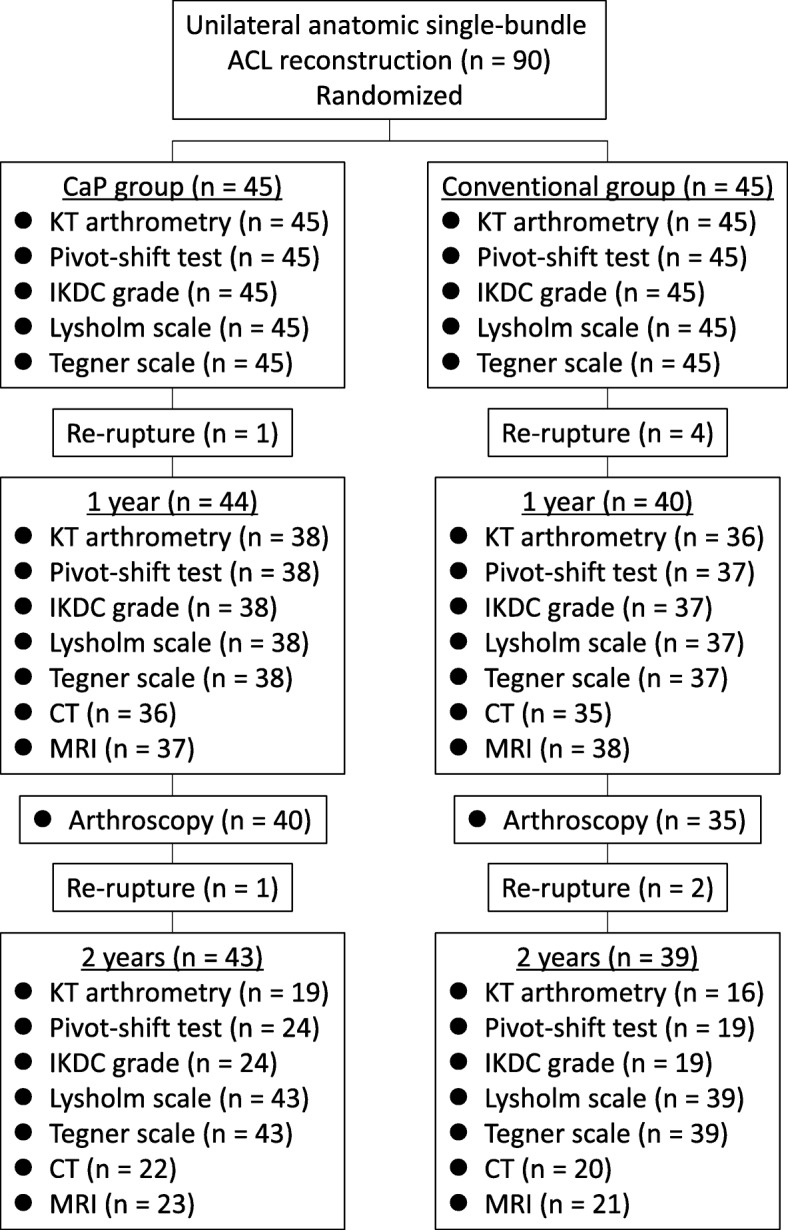
Flowchart of the study. The CaP group underwent single-bundle anterior cruciate ligament reconstruction with a calcium phosphate-hybridized graft, while the conventional group underwent reconstruction with a conventional tendon graft. *ACL* anterior cruciate ligament, *IKDC* International Knee Documentation Committee

A flow-chart of the present study is shown in Fig. 1.

Clinical results are summarized in Table 2. There were two cases of re-rupture in the CaP group (one case occurred within 1 year, and one case occurred more than 1 year postoperatively), and six cases of re-rupture in the conventional group (four cases within 1 year, and two cases after more than 1 year) in 2 years of follow-up. In the two cases of re-rupture in the CaP group, the femoral socket depths were 10 mm and 12 mm, the harvested tendons were semitendinous tendons alone, and the graft diameters were 8 mm and 8.5 mm in femoral side and both 9 mm in tibial side, respectively. The clinical results were evaluated after excluding the re-ruptured cases. Within each group, the anterior knee laxity at 1 and 2 years postoperatively was significantly lesser than preoperatively; there was also a significant difference in pivot-shift test and IKDC results at 1 and 2 years postoperatively compared with preoperatively, and the Lysholm scale at 1 and 2 years postoperatively was significantly larger than preoperatively. There was no significant difference between the CaP and conventional groups regarding anterior knee laxity, pivot-shift test, Tegner scale, IKDC grade, Lysholm scale, and re-rupture rate at the same timepoint. There were no adverse events at both follow-up timepoints in either group.

**Table 2:** Clinical results

	CaP group	Conventional group	*P* value
Re-ruptured cases (*n* (%))			
1 year postoperatively	1 (2.2%)	4 (8.9%)	0.167
2 years postoperatively	1 (2.2%)	2 (4.4%)	0.557
Total	2 (4.4%)	6 (13.3%)	0.138
Anterior knee laxity measured by KT-1000 arthrometry (mm)			
Preoperative	9.2 ± 3.2 (*n* = 45)	10.1 ± 2.5 (*n* = 45)	0.139
1 year postoperatively	0.7 ± 1.1 (*n* = 38) †	0.4 ± 1.0 (*n* = 36) †	0.323
2 years postoperatively	0.9 ± 1.5 (*n* = 19) †	0.1 ± 0.8 (*n* = 16) †	0.094
Pivot-shift test: 0, 1+, 2+, 3+ (*n*)			
Preoperative	0, 8, 37, 0 (*n* = 45)	0, 6, 39, 0 (n = 45)	0.561
1 year postoperatively	36, 1, 1, 0 (*n* = 38) †	35, 2, 0, 0 (*n* = 37) †	0.513
2 years postoperatively	22, 2, 0, 0 (*n* = 24) †	19, 0, 0, 0 (*n* = 19) †	0.198
IKDC grade: A, B, C, D (*n*)			
Preoperative	0, 2, 27, 16 (*n* = 45)	0, 0, 28, 17 (*n* = 45)	0.359
1 year postoperatively	35, 2, 1, 0 (*n* = 38) †	34, 3, 0, 0 (*n* = 37) †	0.548
2 years postoperatively	20, 4, 0, 0 (*n* = 24) †	19, 0, 0, 0 (*n* = 19) †	0.062
Lysholm scale (points)			
Preoperative	54.6 ± 9.6 (*n* = 45)	51.7 ± 7.4 (*n* = 45)	0.117
1 year postoperatively	98.2 ± 2.9 (*n* = 38) †	98.6 ± 2.2 (*n* = 37) †	0.464
2 years postoperatively	98.2 ± 4.5 (*n* = 43) †	99.5 ± 1.8 (*n* = 39) †	0.110
Tegner scale: 0, 1, 2, 3, 4, 5, 6, 7, 8, 9, 10 (*n*)			
Preoperative (preinjury)	0, 0, 0, 2, 3, 3, 18, 10, 1, 8, 0 (*n* = 45)	0, 0, 0, 0, 2, 0, 14, 12, 1, 16, 0 (*n* = 45)	0.201
1 year postoperatively	0, 0, 0, 3, 7, 9, 10, 6, 0, 3, 0 (*n* = 38) †	0, 0, 0, 0, 4, 9, 14, 4, 0, 6, 0 (*n* = 37) †	0.319
2 years postoperatively	0, 0, 0, 3, 2, 6, 14, 10, 1, 7, 0 (*n* = 43)	0, 0, 0, 0, 2, 2, 13, 9, 0, 13, 0 (*n* = 39)	0.260

Results are given as the mean ± SD

Significant difference between the two groups (*P* < 0.05); † significant difference compared with preoperative value (*P* < 0.05)

*IKDC* International Knee Documentation Committee

The CaP group underwent single-bundle anterior cruciate ligament reconstruction with a calcium phosphate-hybridized graft, while the conventional group underwent reconstruction with a conventional tendon graft

Bone tunnel enlargement results are summarized in Table 3. The CSA increase rate in the femoral bone tunnel of the CaP group was significantly smaller than that of the conventional group at 1 year postoperatively. The other results did not significantly differ between groups at either follow-up timepoint.

**Table 3:** Postoperative bone tunnel enlargement

	CaP group	Conventional group	*P* value
Femur			
1 year (%)	24.1 ± 49.8 (*n* = 36)	63.8 ± 66.1 (*n* = 35)	0.006*
2 years (%)	13.4 ± 58.1 (*n* = 22)	41.8 ± 66.7 (*n* = 20)	0.148
Tibia			
1 year (%)	17.3 ± 24.5 (*n* = 36)	18.9 ± 25.5 (*n* = 35)	0.788
2 years (%)	6.4 ± 31.7 (*n* = 22)	1.5 ± 22.5 (*n* = 20)	0.573

Results are given as the mean ± SD

*Significant difference between the two groups (*P* < 0.05)

The CaP group underwent single-bundle anterior cruciate ligament reconstruction with a calcium phosphate-hybridized graft, while the conventional group underwent reconstruction with a conventional tendon graft

The results regarding the intensity of the tendon graft and the arthroscopic appearance are summarized in Table 4. There was no significant difference between groups in the intensity of the tendon graft or the arthroscopic appearance at both follow-up timepoints.

**Table 4:** Postoperative MRI findings and arthroscopic appearance

	CaP group	Conventional group	*P* value
Graft intensity on MRI: low, iso, high			
1 year	20, 17, 0 (*n* = 37)	16, 21, 1 (*n* = 38)	0.396
2 years	16, 6, 1 (*n* = 23)	19, 2, 0 (*n* = 21)	0.205
Arthroscopic appearance: A, B, C	25, 12, 3 (*n* = 40)	22, 9, 4 (*n* = 35)	0.806

Significant difference between the two groups (*P* < 0.05)

The CaP group underwent single-bundle anterior cruciate ligament reconstruction with a calcium phosphate-hybridized graft, while the conventional group underwent reconstruction with a conventional tendon graft

The arthroscopic appearance of the synovium coverage of each graft was graded as A (completely covered), B (partially covered), or C (almost uncovered)

## Discussion

The main findings of the present study were that anatomic single-bundle ACL reconstruction using the CaP-hybridized tendon graft was safe, resulted in improved clinical results at 1 and 2 years postoperatively compared with preoperatively, and was as stable as the conventional method. The femoral bone tunnel increase rate at 1 year postoperatively in the CaP group was smaller than that in the conventional group. The postoperative intensity of the tendon graft and arthroscopic appearance in the CaP group were equivalent to those in the conventional group. The CaP hybridization technique is simple, and can be applied any patients ACL reconstruction is required. Moreover, the cost is inexpensive because it only involves the solutions preparation.

The postoperative clinical results in the CaP group were improved compared with preoperatively, and were similar to the results after conventional anatomic single-bundle ACL reconstruction. Moreover, no adverse events occurred in the follow-up period. Therefore, the CaP-hybridized tendon graft safely and effectively improved the postoperative clinical results in anatomic single-bundle ACL reconstruction. In non-anatomic single-bundle ACL reconstruction, the clinical results at 1 and 2 years postoperatively in the CaP group were reportedly superior to the results after reconstruction via the conventional method [7]. However, in the present study, the clinical results at 1 and 2 years postoperatively were equal in both groups. This may be because anatomic ACL reconstruction results in superior stability compared with non-anatomic ACL reconstruction [16, 17], and the follow-up period was short in the present study. Therefore, a longer follow-up is needed to clarify the clinical effects of the CaP-hybridized tendon graft in anatomic single-bundle ACL reconstruction.

In the present study, the CaP-hybridized tendon graft resulted in a reduced femoral bone tunnel enlargement compared with the conventional method at 1 year postoperatively; the lack of data at 2 years postoperatively in both groups may be the reason that no significant difference was detected between groups at 2 years. The articular ends of the bone tunnels are enlarged by bone resorption, which is associated with graft-tunnel motion after ACL reconstruction with suspensory fixation [18, 19], and synovial fluid cytokines cause delayed tendon-to-bone healing at the joint aperture site of the bone tunnel [20]. The CaP-hybridized tendon graft enhances bone formation on the surface of the tendon graft in the bone tunnel compared with the untreated tendon graft [5, 6]. Therefore, more new bone can form in the femoral tunnels in the CaP method than the conventional method because of its osteogenic effect. Previous studies in the goat revealed that the CaP-hybridized tendon graft enhanced tendon-to-bone healing in the cartilage layer, and enhanced the new formation of bone near the joint aperture of the femoral and tibial bone tunnels [3, 8]. The cartilage layer can act as a shock absorber against graft-tunnel motion, and as a protective wall against synovial fluid when it is anchored near the joint aperture. The anchoring provided by new bone formation and the cartilage layer in the CaP group may effectively prevent the progression of bone tunnel enlargement. Longer follow-up is needed to investigate the bone tunnel enlargement, and the association between bone tunnel enlargement and knee stability. In the present study, the CaP method prevented bone tunnel enlargement, especially on the femoral side. Bone tunnel enlargement was particularly evident on the femoral side in conventional ACL reconstruction in a previous study [21]. Greater shear stress can occur at the interface in the femoral bone tunnel than on the tibial side, comprising both longitudinal micromotion (bungee effect) and transverse micromotion (windshield wiper effect) [22].

The tendon grafts in the two groups in the present study had a similar intensity on MRI and a similar arthroscopic appearance. The CaP hybridization method did not cause complications in the grafted tendons on MRI and arthroscopic analysis. This indicates that the CaP-hybridized tendon graft is safe for use in clinical practice. The intensity of tendon grafts on MRI and the arthroscopic appearance of the tendon grafts indicate the graft maturation process (ligamentization and revascularization) after ACL reconstruction [23, 24]. We believe that firm anchoring is important for maturation of the tendon graft, as firm anchoring can lead to the application of appropriate mechanical stress on the tendon graft. MRI analysis at more than 2 years after ACL reconstruction may be required to more accurately evaluate the ligamentization and revascularization of the tendon grafts in the two groups.

The present study had some limitations. The evaluations were only performed at three timepoints, and the follow-up period was short. Furthermore, there were missing data. Longer follow-up with a complete dataset is needed to investigate any increase in instability, bone tunnel enlargement, and graft maturation. Moreover, it is necessary to evaluate the relationship between bone tunnel enlargement, graft maturation, and knee instability.

## Conclusions

Anatomic single-bundle ACL reconstruction using the CaP-hybridized tendon graft is safe, and results in improved postoperative clinical results compared with preoperative data during 2 years of follow-up. The postoperative clinical results in the CaP group were as stable as those in the conventional group. The CaP-hybridized tendon graft can reduce postoperative femoral bone tunnel enlargement compared with the untreated tendon graft. A longer follow-up is needed to clarify the clinical effects of the CaP-hybridized tendon graft in anatomic single-bundle ACL reconstruction.

## Abbreviations

ACL: Anterior cruciate ligament
CaP: Calcium phosphate
CSA: Cross-sectional area
CT: Computed tomography
IKDC: International Knee Documentation Committee
MRI: Magnetic resonance imaging
